# Electrophysiology Measurements of Metal Transport by MntH2 from *Enterococcus faecalis*

**DOI:** 10.3390/membranes10100255

**Published:** 2020-09-24

**Authors:** Matthias Gantner, Theodoros Laftsoglou, Honglin Rong, Vincent L. G. Postis, Lars J. C. Jeuken

**Affiliations:** 1 School of Biomedical Sciences and the Astbury Centre for Structural Molecular Biology, University of Leeds, Leeds LS2 9JT, UK; matthiasgantner@hotmail.com (M.G.); t.laftsoglou@gmail.com (T.L.); H.Rong@leeds.ac.uk (H.R.); 2 School of Clinical & Applied Sciences, Leeds Beckett University, Leeds LS1 3HE, UK; V.L.G.Postis@leeds.ac.uk

**Keywords:** transporters, metal, manganese, solid-supported membranes, electrophysiology

## Abstract

Transition metals are essential trace elements and their high-affinity uptake is required for many organisms. Metal transporters are often characterised using metal-sensitive fluorescent dyes, limiting the metals and experimental conditions that can be studied. Here, we have tested whether metal transport by *Enterococcus faecalis* MntH2 can be measured with an electrophysiology method that is based on the solid-supported membrane technology. *E. faecalis* MntH2 belongs to the Natural Resistance-Associated Macrophage Protein (Nramp) family of proton-coupled transporters, which transport divalent transition metals and do not transport the earth metals. Electrophysiology confirms transport of Mn(II), Co(II), Zn(II) and Cd(II) by MntH2. However, no uptake responses for Cu(II), Fe(II) and Ni(II) were observed, while the presence of these metals abolishes the uptake signals for Mn(II). Fluorescence assays confirm that Ni(II) is transported. The data are discussed with respect to properties and structures of Nramp-type family members and the ability of electrophysiology to measure charge transport and not directly substrate transport.

## 1. Introduction

Transition metals are essential micronutrients for most organisms, including many bacterial pathogens, and one of the host immune strategies is to starve phagocytosed microorganism from transition metal micronutrients [1,2]. A transition metal transporter expressed in the phagosomal membranes of macrophages, SLC11A1, is thought to reduce the metal bioavailability to pathogens as part of the immune defence system [3,4,5]. This transporter belongs to the Natural Resistance-Associated Macrophage Protein (Nramp) family. Members of this family are proton-coupled transporters of divalent transition metals that do not transport the earth metals Ca(II) and Mg(II). They function as symporters or secondary active transporters and utilise the driving force of proton and electrochemical gradients to transport their substrates from a low extracellular (or phagosomal lumen) concentration to a higher intracellular concentration.

In Vitro studies of metal transport across lipid membranes often make use of fluorescent dyes such as calcein that quench upon metal binding. However, metal binding by these dyes is often pH dependent, while quenching and metal affinity varies between transition metals. This can limit the type of experiments that can be performed or the experimental conditions that can be investigated. An alternative method to study charge transport across lipid membranes is electrophysiology based on a solid-supported membrane platform. In this setup, developed in the 1990s by Fendler and co-workers [6,7], proteoliposomes are adsorbed on a modified gold sensor connected to a picoampere amplifier within a flow system. In this system, transport of charged species across the membrane leads to a charge redistribution close to the gold sensor, which is detected by a transient electric current. Here, we have tested whether this electrophysiology method can be used to measure metal transport using the manganese transporter MntH2 from *Enterococcus faecalis* as a model system. MntH2 belongs to the Nramp family of transporters and plays a key role in virulence of *E. faecalis*, which causes some of the most important hospital-acquired, drug-resistance infections, including urinary tract infections (UTIs), endocarditis and sepsis [8,9,10].

Bacteria express a variety of different manganese transporter classes: (i) ABC transporters, (ii) Nramp-type symporters or, less common, (iii) P-type ATPases [2]. Deletion of the Mn(II) ABC transporters attenuates or completely abolishes virulence in *Mycobacterium tuberculosis*, *Bacillus anthracis* and various streptococci, yersiniae and salmonellae [11]. Intracellular replication of *Salmonella enterica* in macrophages is attenuated when both the ABC transporter (*sitABCD*) and the Nramp symporter (*mntH*) are mutated [12]. Similarly, in *Staphylococcus aureus,* both transporters (*mntABC* and *mntH*) need to be deleted before a reduction in the number of bacteria is observed in a murine infection model [13]. The *Enterococcus faecalis* genome bears three Mn(II) transporters, one ABC transporter (*efaCBA*) and two Nramp-type symporters (*mntH* and *mntH2*). The deletion of *efaCBA* and *mntH2* is sufficient to abolish virulence in mammalian models [14].

**Figure 1:**
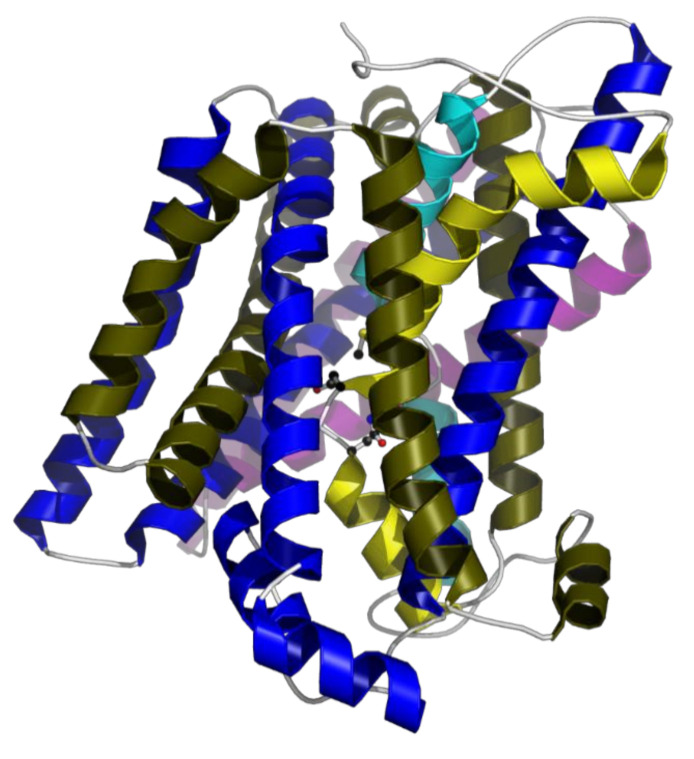
Structural model of MntH2 from *E. faecalis*. The model has been prepared with I-TASSER and is mostly based on the structure of *E. coleocola* MntH (PDB ID: 5M87) [20]. The yellow and blue helices relate to the two related halves of the Nramp family, which shares features with the LeuT transporter family. Yellow and blue refer to helix 1–5 and helix 6–10, respectively. Light yellow and light blue designate the kinked helices 1 and 6. Magenta helices are helix 11 and 12. Only residues 21 to 514 of the protein model are shown. The four residues coordinating to the divalent metals are shown in ball-and-stick format. Image made with Molscript.

MntH2 from *E. faecalis* has a high homology with MntH from *Eremococcus coleocola* (67% identity) for which a structure has been solved [15]. Secondary structure analysis and structural modelling shows that *E. faecalis* MntH2 has 12 transmembrane helices and all functionally important residues, including those in the substrate-binding pocket, are conserved (Figure 1). Structural characterisation of bacterial Nramp homologues from *E. coleocola, Staphylococcus capitis* and *Deinococcus radiodurans* indicate that these transporters operate *via* an alternating-access mechanism, which is common to uniporters and symporters [15,16,17,18]. These structures display a LeuT-like fold which is likely to be shared among Nramp family members. The residues of the metal-binding pocket are an alanine (backbone oxygen), an asparagine, an aspartate and a methionine (Ala239, Asp59, Asn62 and Met242 in *E. faecalis*). It has been demonstrated that the methionine plays a key role in distinguishing between transition metal and earth alkali metal ions [19]. Gaudet and colleagues have shown that substitution of this methionine by alanine in *D. radiodurans* MntH led to a fully functional mutant; but, in addition to transition metals, this mutant can transport calcium and magnesium ions [19]. As expected from a LeuT fold, the metal substrate-binding site is located in the unwound part of transmembrane helices 1 and 6 and this site if fully conserved in MntH2 from *E. faecalis* (Figure 1).

## 2. Materials and Methods 

### 2.1. Expression and Purification

The design and cloning of the expression vector (pL33-MntH2) for *E. faecalis* MntH2 (accession number GenBank: VFU79923.1) has been described previously [21]. MntH2 is expressed with an octa-histidine affinity tag (RGSH_8_ tag) at the C-terminus preceded by a HRV-3C protease cleavage site. The expression vector is based on pTTQ18, in which expression is under the control of the tac promoter [22]. Overexpression of MntH2 in BL21*/pRARE/pL33-MntH2 was performed in SB autoinduction medium (supplemented with 100 µg/mL carbenicillin) for 22–24 h at 37 °C, 230 rpm in 500 mL batches in 2 L baffled flasks. Cell were harvested at 7000× *g* for 15 min. Typically, 80 g of wet cell paste was obtained from a 4 L culture medium. 

Purification was performed using a modified protocol to that published previously [21]. Cells were resuspended at 4 °C in lysis buffer (100 mM HEPES, pH 7.0, 150 mM NaCl) and after addition of 0.5 mM Phenylmethanesulfonyl fluoride (PMSF) from an ethanol stock, lysed by two passages through a cell disruptor (Constant Systems Ltd., Daventry, UK) at 30 kpsi. Debris were removed by centrifugation (30 min at 12,000× *g*). The supernatant was further centrifuged at 100,000× *g* for 60 min to harvest the membranes, which were resuspended in a small amount of lysis buffer. After determining the protein concentration using a BCA assay (Thermo-Fisher, UK) according to the manufacturer’s protocol, the membranes were solubilised by dilution in solubilisation buffer (20 mM HEPES, pH 8.0, 300 mM NaCl; 5% (*v*/*v*) glycerol; 1.5% (*w*/*v*) n-Dodecyl β-D-maltoside (DDM), 10 mM Imidazole) for 2 h at 4 °C at a concentration of 3 mg/mL total protein. Insoluble material was removed by centrifugation at 100,000× *g* for 1 h. The solubilised membrane proteins were loaded on a Ni-NTA column (HisTrap HP, 5 mL GE Healthcare, UK), pre-equilibrated with wash buffer (25 mM HEPES, pH 7.0, 300 mM NaCl, 5% glycerol, 0.05% DDM) with 10 mM imidazole, using a Äkta pure (GE Healthcare, UK) at 0.5 mL/min. After washing the Ni-NTA column with 50 mL of wash buffer containing 80 mM imidazole, the protein was eluted using a linear gradient in wash buffer from 80 to 400 mM imidazole over 80 mL at 0.5 mL/min. The protein elutes at ~200 mM imidazole. The eluate is concentrated using 30 kDa cut-off centrifugal concentrators (VivaSpin, Vivaproducts, USA) and dialysed against dialysis buffer (20 mM MES, pH 6.0, 150 mM NaCl, 5% glycerol, 0.05% DDM) for 2 days with 2 changes of buffer. SDS-PAGE showed a minor impurity at ~7 kDa, which was removed by size-exclusion chromatography (Superdex S200, GE Healthcare, UK at 0.5 mL/min; running buffer identical to dialysis buffer). Mass spectrometry confirmed the identity of MntH2. For SDS-PAGE images shown in the result section, brightness and contrast (uniformly over the whole image) were enhanced using PaintShop Pro to improve clarity. 

Some experiments were performed with MntH2 from which the his-tag was cleaved using HRV-3C protease. An N-terminally octahistidine-tagged form of HRV-3C protease in a pET28 vector was expressed in BL21*(DE3)/pRARE in a SB autoinduction medium at 30 °C for 22–24 h and purified using Ni-NTA affinity chromatography. Typically, purified MntH2 was incubated with HRV-3C at a 1:2 weight ratio in 20 mM HEPES, pH 8.0, 300 mM NaCl, 10 mM imidazole, 10% glycerol, 0.05% DDM. Following a 6–24 h incubation at 4 °C, the solution was loaded on a Ni-NTA column to remove the protease and the cleaved tag. MntH2 free from its tag was collected in the flow-through. As described in the Result section, no difference in signals was observed in the electrophysiology experiments between MntH2 with or without the his-tag. 

### 2.2. Reconstitution of MntH2 and Fluoresce Uptake Assay

*E. coli* ‘polar’ lipids (Avanti Lipids) were aliquoted in 5 mg batches in glass vials from a chloroform stock solution and solvent was removed by desiccation under vacuum for 2 h. Lipids were rehydrated in 10 mM HEPES, pH 7.0, 150 mM NaCl at 5 mg/mL by vortex mixing. Liposomes were formed by extrusion through 200 nm track-etched polycarbonate membranes (Avanti extruder, Avanti, USA) using the manufacturer’s protocol. DDM was added to a final concentration of 1% (*w*/*v*) in 4 mg/mL lipids and MntH2 was added from a > 1 mg/mL stock to the desired lipid-to-protein ratio (LPR is given in *w*/*w* and is typically 10:1). The solution was incubated on ice for 15 min and DDM was subsequently removed with biobeads SM2 (Biorad Laboratories Ltd, UK). Biobeads were consecutively added four times (0.07, 0.07, 0.14 and 0.14 g/mL) and incubated on ice respectively for 30, 60, 60 and 120 min. Proteoliposomes were aliquoted, snap-frozen in liquid nitrogen and stored at −80 °C until use. Liposomes without MntH2 were prepared using the same protocol for control experiments.

For experiments with calcein or 8-hydroxypyrene-1,3,6-trisulfonic acid (HPTS), the same procedure was followed, but the lipids were resuspended in 5 mM MOPS, pH 7.0, 150 mM KCl with either 250 µM calcein or 1 mM HPTS. After proteoliposome formation, non-encapsulated dye was removed by dialysis for 36 h with two changes of buffer (5 mM MOPS, pH 7.0, 150 mM KCl). Liposomes were diluted to 0.04 mg lipid/mL for calcein or 0.5 mg lipid/mL for HPTS encapsulated liposomes (assuming no lipids were lost in the reconstitution procedure).

Fluorescence quenching of calcein due to metal binding was monitored in a quartz cuvette (excitation 490 nm, emission at 515 nm). The rate of uptake was determined using the slope of fluorescence decrease over the first 20 s. Michaelis–Menten parameters were determined by fitting the uptake rate as a function of metal concentration against a Michaelis–Menten model using non-linear regression (two free parameters: υ_max_ and K_M_^app^).

The pH variation of the lumen was monitored by measuring the ratio of emission at 510 nm by HPTS with excitation at 400 and 448 nm in a quartz cuvette. The ratiometric HPTS fluorescence data were converted to pH values using a calibration curve measured for 0.5 μM HPTS between pH 6 and 8.

### 2.3. Electrophysiology Experiments

Electrophysiology experiments based on solid-supported membrane technology was performed on a Nanion SURFE^2^R N1 workstation using the manufacturer’s sensors. MntH2 proteoliposomes stock solutions (4 mg/mL lipids) were diluted 50 times in non-activating buffer B (25 mM HEPES/NaOH, pH 7.0, 150 mM NaCl, 2 mM MgCl_2_) and sonicated for 10 pulses (UPH50 sonicator, Hielscher GmBH, tip-size = 1 mm, Amplitude = 0.5, cycle = 0.2). SURFE^2^R sensors were prepared according to the manufacturer’s protocol with slight modifications. Sensors were incubated in 0.1 mM 1-octadecanethiol in 50:50 isopropanol/water for 15 min. Sensors were rinsed three times with isopropanol and dried under a nitrogen gas flow. A volume of 3 µL of 7.5 mg/mL 1,2 diphytanoyl-phosphatidylcholine in decane was ‘dropped’ onto the sensor surface followed by immediate addition of 50 µL buffer B and incubated for 30–60 min at 4 °C. The buffer/lipid/decane solution was removed from the sensor and 100 µL of the MntH2 proteoliposome solution was added immediately. Sensors were centrifuged at 800× *g* for 45 min at 4 °C and installed in the SURFE^2^R N1 workstation. Sensors were used within three days and stored at 4 °C when not in use. Electrophysiology experiments were performed at 20 °C and typically consisted of a three phase solution exchange protocol at 250 µL/s: (1) non-activating buffer B (1 s), (2) activating buffer A (buffer B with transition metal at given concentration; 1 s), and (3) non-activating buffer B (1 s). Occasionally, the first stage (1) was extended to 2 s. Each measurement was repeated at least four times (n ≥ 4), and with at least two different sensors (N ≥ 2). Current traces are corrected for small offset differences (< 50 pA). Experiments were typically performed at pH 8.0 (10 mM HEPES/NaOH, pH 8.0, 150 mM NaCl, 2 mM MgCl_2_ or 10 mM HEPES/KOH, pH 8.0, 150 mM KCl, 2 mM MgCl_2_). Metals were always added from a metal-chloride solution (e.g., MnCl_2_). In some experiments with FeCl_2_, 1 mM ascorbic acid was added to prevent oxidation of Fe(II). No difference was observed between experiments with and without ascorbic acid.

## 3. Results

**Figure 2:**
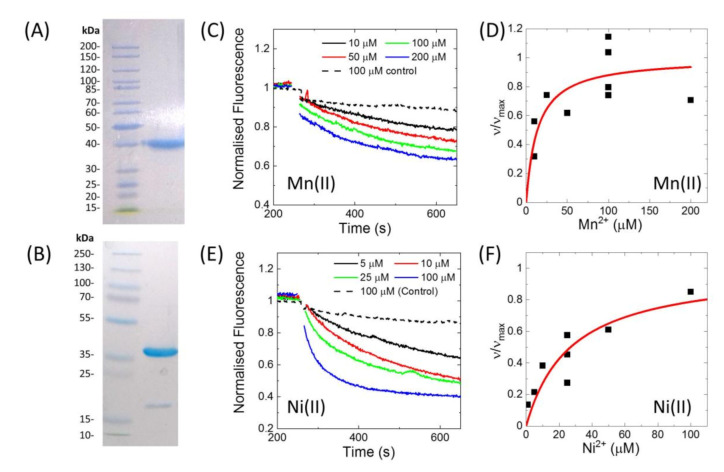
(**A**) SDS-PAGE gel of the purified, his-tagged MntH2. (**B**) SDS-PAGE gel of MntH2 after the his-tag was removed by treatment with HRV-3C protease. The impurity at ~18 kDa is believed to be HRV-3C protease, which was not completely removed by IMAC chromatography. (**C**,**E**) Concentration-dependent quenching of calcein in metal uptake assays. Calcein dye was encapsulated into proteoliposomes with his-tagged MntH2 at LPRs of (**C**,**D**) 70:1 or (E, F) 110:1 (*w*/*w*). Metals were added at ~250 s. The dashed lines are control experiments using liposomes without MntH2. (**D**,**F**) Initial velocities of metal uptake were determined by a linear fit of the first 20 s of the traces measured in three different MntH2 proteoliposomes samples and plotted against metal concentration (black squares) and fitted with a Michaelis–Menten model using non-linear regression (red line). The uptake rates (υ) are normalised against maximum uptake rates (υ_max_) determined from the fit. The K_M_^app^ for Mn(II) and Ni(II) was ~15 µM. Experiments are performed in 5 mM MOPS, pH 7.0, 150 mM KCl.

His-tagged MntH2 from *E. faecalis* was purified after heterologous expression in *E. coli* using affinity chromatography and size-exclusion chromatography. Figure 2A show an SDS-PAGE gel of the purified protein, indicating a purity of >95%. The his-tag could be removed by specific proteolytic cleavage of the HRV-3C protease site present in between the his-tag and MntH2 (Figure 2B).

Activity of the purified MntH2 in proteoliposomes was confirmed by a fluorescent assay with calcein-loaded proteoliposomes (LPR 70:1 to 110:1, *w*/*w*) using a similar assay as reported for Nramp-type transporters from *S. capitis* and *D. radiodurans* [16,17]. Figure 2 shows Mn(II) and Ni(II) transport traces and using the initial rate of metal uptake, a K_M_^app^ was estimated to be approximately 20 µM for both Mn(II) and Ni(II). In this assay, calcein is quenched by Mn(II), Ni(II) and Cu(II). When calcein was encapsulated in control liposomes (without MntH2), it was still quenched by Cu(II), hence Cu(II) transport by MntH2 could not be monitored using this assay. We note that this is not due to liposomes leakage or dye bound to the outside of the liposomes as Mn(II) and Ni(II) do not show quenching of calcein in control liposomes (Figure 2C,E). Although we cannot determine the reason why calcein is quenched in our control experiments, ‘unassisted’ Cu(II) transport into liposomes has been observed when liposomes are loaded with metal chelators [23].

**Figure 3:**
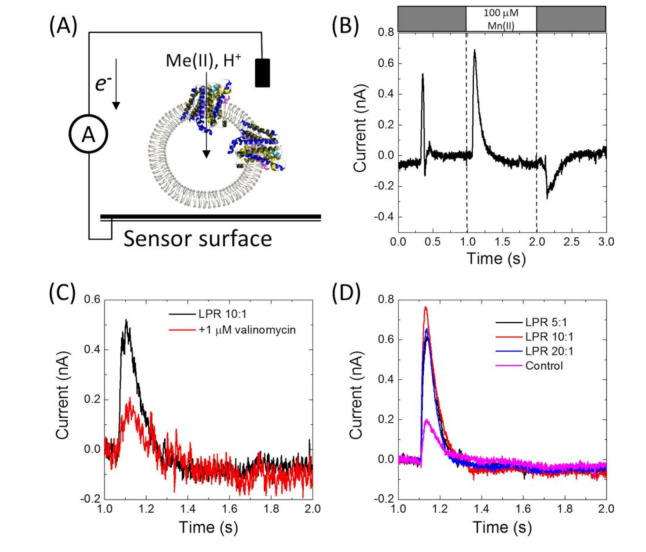
(**A**) Schematic representation of the electrophysiological method based on a solid-supported membrane platform. Proteoliposomes are adsorbed on a gold sensor surface. Transport of charges species into the lumen of the liposomes results in a current in the electric circuit between the solution and the gold surface. (**B**) Example of the electrophysiology experiments of his-tagged MntH2 proteoliposomes (LPR 10:1 (*w*/*w*)) in 10 mM MES/HEPES/CHES buffer, pH 8.0, 140 mM KCl and 2 mM MgCl_2_. Between 1 and 2 s, 100 µM MnCl_2_ was used to initiate uptake. (**C**) The same experiment as in (B) in 10 mM HEPES buffer, pH 8.0, 140 mM KCl and 2 mM MgCl_2_ before and after addition of 1 μM valinomycin. (**D**) The same experiments as (B) in 10 mM HEPES, pH 8.0, 150 mM KCl, 2 mM MgCl_2_ at different LPR (*w*/*w*), but using MntH2 from which the octa-histidine tag has been removed. The control experiment was performed using liposomes prepared in an identical way to the MntH2 proteoliposomes, but without MntH2.

Having confirmed that MntH2 is active using ‘classic’ fluorescent quenching assays, it was tested whether metal transport could be measured using an electrophysiology method based on a solid-supported membrane platform (Figure 3A). Figure 3B shows a typical result for MntH2 proteoliposomes. A constant flow of buffer solution (without transition metals) is started at ~0.25 s, resulting in a non-specific transient current signal, which returns to the baseline within a second. Without disrupting the flow, at ~1 s, the system switches to a buffer containing substrate, in this case 100 μM Mn(II), resulting in the uptake of metals and possibly protons into the vesicles and a transient charge redistribution at the gold sensor’s surface. The latter is detected as an electric current, typically ~0.6 nA for 100 μM Mn(II), at approximately ~1 s. We found that peak currents were consistent between experiments performed with a single sensor, but variations in peak currents were observed between different sensors (between 0.3 and 0.8 pA).

The current is directly proportional to the uptake kinetics of the transporter and hence an almost instant rise in current is detected when Mn(II) is supplied. This instant rise is followed by a slower decrease in current. For reasons explained below, we propose that the current returns to baseline because the Mn(II) uptake reaches a thermodynamic equilibrium. At ~2 s, the buffer in the flow system switches back to the buffer without substrate and the transport of Mn(II) out of the vesicles results in a less well defined opposite current peak signal.

Metal binding to the surface of proteoliposomes would lead to a background signal in a similar way to metal transport (see Figure 3A). We minimized this potential artefact by inclusion of 2 mM MgCl_2_ to all buffers. Although some background signals remained and were found to vary between sensors, they were typically ~0.2 nA for 100 μM Mn(II) (Figure 3D; the control sample is a sensor prepared with liposomes without MntH2). Uptake signals for MntH2 proteoliposomes could be abolished by the addition of 1 μM valinomycin, leaving only the background signals (Figure 3C). This further confirms that the signals are due to metal transport and not due to metal binding at the outside surface of the liposomes.

MntH2 is purified by Immobilized Metal Affinity Chromatography (IMAC) using a C-terminal octa-histidine tag. To further ensure that the signals do not arise from binding of Mn(II) to the histidine tag, experiments were also performed with MntH2 samples from which the tag was removed. Similar peak currents were obtained in the presence or absence of the tag, Figure 3B,D respectively. Figure 3D also shows that Mn(II) uptake signals for proteoliposomes prepared with different LPRs (1:5, 1:10 and 1:20, *w*/*w*) are not significantly different. Therefore, the remainder of the experiments were performed at an LPR of 10:1.

**Figure 4:**
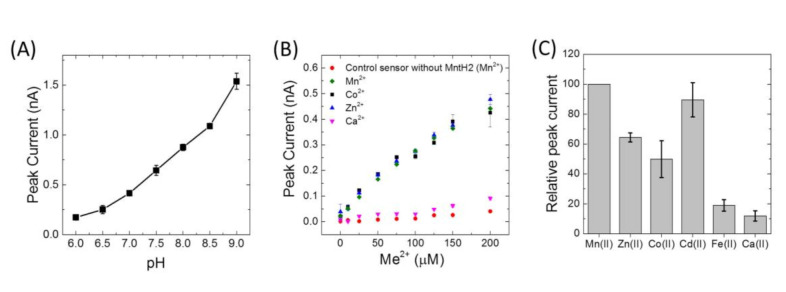
(**A**) Electrophysiology peak currents as a function of pH for MntH2 proteoliposomes (LPR 10:1 (*w*/*w*)) with MntH2 from which the octa-histidine tag was removed. Buffer was 5 mM MES/HEPES/CHES, 150 mM KCl, 2 mM MgCl_2_ Peak currents are obtained with 100 μM Mn(II) and corrected for background currents, which were obtained by measuring electrophysiology traces in the presence of 1 μM valinomycin. (**B**) Peak currents recorded from a single sensor in 10 mM HEPES, pH 8.0, 150 mM KCl, 2 mM MgCl_2_ with his-tagged MntH2 proteoliposomes (LPR 10:1 (*w*/*w*)) as a function of the tested divalent metal (Me^2+^), as indicated. The control experiment was performed on a different sensor prepared with liposomes without MntH2. (**C**) Relative peak currents obtained with 100 μM of the indicated metals for four different sensors in 10 mM HEPES, pH 8.0, 150 mM KCl, 2 mM MgCl_2_ with his-tagged MntH2 proteoliposomes (LPR 10:1 (*w*/*w*)). The data between sensors are normalised for the peak current measured with 100 μM Mn(II). For Fe(II), 1 mM of ascorbic acid was added to prevent oxidation to Fe(III).

**Figure 5:**
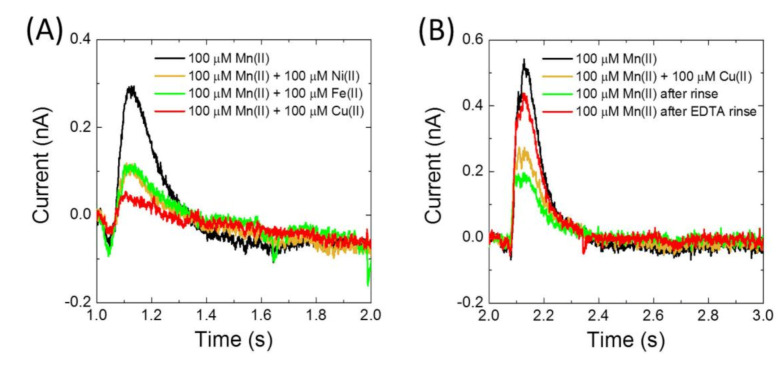
(**A**) Electrophysiology experiments of his-tagged MntH2 proteoliposomes (LPR 10:1 (*w*/*w*)) in 10 mM HEPES, pH 8.0, 150 mM NaCl, 2 mM MgCl_2_. Between 1 and 2 s, 100 µM MnCl_2_ was used to initiate uptake either without other metals or together with 100 µM of Fe(II), Ni(II) or Cu(II) as indicated. (**B**) Same as (**A**), but metal uptake of Mn(II) was initiated between 2 and 3 s in the absence and then presence of 100 µM of Cu(II). After exposing MntH2 to Cu(II), the sensor cell was rinsed intensively with buffer, but Mn(II) uptake was not recovered, unless the cell was rinsed with 10 mM EDTA solution.

Peak currents of Mn(II) uptake were observed to increase by approximately 4 fold from pH 6 to 9 (Figure 4A). Because of the higher signals at alkaline pH, metal specificity was studied at pH 8.0. Similar transport activities were obtained for Mn(II), Co(II), Zn(II), Cd(II), but no uptake was detected for Ca(II), Fe(II), Cu(II) or Ni(II) (Figure 4B,C). Importantly, the presence of Fe(II), Cu(II) and Ni(II) was observed to impair current signals due to uptake of Mn(II) (Figure 5). In fact, after using Cu(II), activity of MntH2 could only be recovered if the sensor was rinsed with a solution containing a strong metal chelator, such as ethylenediaminetetraacetic acid (EDTA) (Figure 5B).

**Figure 6:**
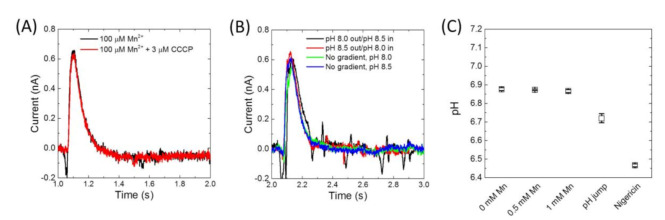
(**A**) Electrophysiology experiments of his-tagged MntH2 proteoliposomes (LPR 10:1 (*w*/*w*)) in 10 mM HEPES, pH 8.0, 150 mM KCl and 2 mM MgCl_2_ in the presence and absence of 3 μM CCCP. Mn(II) was initiated by the addition of 100 μM MnCl_2_. (**B**) Same as (**A**), except some experiments were performed in the presence of a pH gradient. pH gradients were formed by (black line) exchanging the sensor buffer of pH 8.5, with a buffer of pH 8.0 at t = 0 s, followed by the addition of 100 μM Mn(II) at t = 2 s also in pH 8.0. A similar, but opposite sequence was used for the red line. The pH gradient had to be formed prior to adding Mn(II) as changing pH also produces transient electrical currents. (**C**) Fluorescence experiments of HPTS encapsulated his-tagged MntH2 proteoliposomes (LPR 70:1 (*w*/*w*)) in 5 mM MOPS, pH 7.0, 150 mM KCl. HPTS was used to measure the lumen pH of the proteoliposomes after consecutively adding 0.5 mM MnCl_2_, 1 mM MnCl_2_, 0.5 mM HCl (which lowers the pH to ~6.5) and 0.1 μM nigericin. Each addition and measurement are ~2 min apart. The fact that the lumen pH does not immediately equilibrate fully after addition of 0.5 mM HCl indicates that proteoliposomes are able to maintain a pH gradient on this timescale.

As a member of the Nramp family, MntH2 is assumed to be a Me^2+^:H^+^ symporter. The divalent metals together with the proton uptake in the proteoliposome should therefore contribute to the measured currents in Figure 3 and Figure 4. To confirm the contribution of protons to the divalent metal transport by MntH2, electrophysiological measurements were performed in the presence of a typical proton uncoupler, carbonyl cyanide m-chlorophenylhydrazone (CCCP). As CCCP enables a rapid proton translocation, the intensity of the measured current should be affected by the presence of a proton uncoupler [24]. However, as shown in Figure 6A, the measured signal is unperturbed by the addition of CCCP to the sensor. This result suggests that MntH2 is not a proton symporter under these conditions (pH 8.0 in the absence of the transmembrane proton gradient, ΔpH). Furthermore, no significant differences in uptake signals were observed in presence or absence of a ΔpH (Figure 6B), other than small changes expected due to the pH dependence. To create a ΔpH across the membrane, we took advantage of the electrophysiology experimental setup. In these experiments, a ΔpH is created by raising or lowering the pH of the buffer solution by 0.5 units at time 0 s, followed by adding Mn(II) 2 s later. For instance, the system is first equilibrated at pH 8.0 for > 5 min to ensure the lumen pH is at 8.0. At time 0, the buffer is replaced by a buffer at pH 8.5, creating a ΔpH of 0.5. Two seconds later, before ΔpH has dissipated, Mn(II) is added in the pH 8.5 buffer. We note that we cannot change the pH of the buffer simultaneously with the addition of Mn(II), as changes in buffer composition (i.e., pH changes) lead to background signals in this electrophysiology setup.

Electrophysiology experiments were repeated either in sodium or potassium buffers, as well as in buffers with and without chloride, but no differences were observed (not shown). Together, the electrophysiology data suggest that MntH2 is not a proton or sodium symporter at pH 8.0 and that chloride does not contribute to transport. To exclude that this behaviour is due to the electrophysiology setup, solution experiments were performed with proteoliposomes loaded with a pH-dependent fluorescent dye, 8-hydroxypyrene-1,3,6-trisulfonic acid (HPTS). HPTS is a ratiometric dye where the ratio of fluorescence at 510 nm from excitations at 448 nm and 400 nm is used to determine the lumen pH. Addition of up to 1 mM Mn(II) to HPTS-loaded MntH2 proteoliposomes does not cause a detectable shift of pH inside the vesicle (Figure 6C). To confirm that the proteoliposomes were not leaky to protons, the outside pH was, subsequently, lowered by 0.5 units (Figure 6C, “pH jump”), which confirms the proteoliposomes could maintain a ΔpH on the timescale of this experiment (minutes). Finally, the ΔpH is dissipated by the addition of nigericin after which the lumen pH equilibrates with that of the extravesicular buffer (Figure 6C, “nigericin”). Together with electrophysiological experiments, these results strongly suggest that the transport of metal by MntH2 does not involve the co-transport of protons at pH 8.0.

## 4. Discussion

Electrophysiology of solid-supported membranes monitors all electrogenic processes that are related to transport activity, which can include substrate binding, conformational change and transport [7]. We can exclude the possibility that the electrophysiology signals are due to metal binding to either MntH2 or the liposomes, because peak currents are inhibited by either Ni(II), Fe(II) or Cu(II), two ions that on their own do not give rise to a signal. Inhibition by Ni(II), Fe(II) and Cu(II) is very likely to be competitive as structures of the MntH2 homologue *S. capitis* show that Ni(II), Fe(II) and Cu(II) bind to the same conserved binding pocket as Mn(II) [16]. We note that it cannot be excluded that the electrophysiological signals are caused by a metal-induced conformational change of MntH2 (without the metal substrate being transported), but the latter seems unlikely as conformational changes of transporters are typically an integral part of their transport mechanism. We thus conclude that the electrophysiology is able to monitor metal transport by secondary active transporters and the current signals directly correspond to the transport of divalent metal ions.

Changing the LPR ratio does not affect the electrophysiology measurements, indicating that Mn(II) uptake peak currents are not limited by the number of MntH2 proteins in each liposomes. We propose that this is due to the low lumen volume of the proteoliposomes and the effect that Mn(II) uptake has on the electrochemical gradient across the membrane. Assuming proteoliposomes are 200 nm in diameter, it can be estimated that transport of 250 Mn(II) atoms into the lumen would already create a intravesicular concentration of 100 µM. For the LPRs used in this work, in the order of hundreds of transporters are present per liposome and hence a single turnover by MntH2 might already cause a thermodynamic equilibrium. In other words, the peak currents observed do not reflect uptake kinetics, but a thermodynamic limit in uptake. This hypothesis is supported by electrophysiology experiments with different concentrations of Mn(II), which show an almost linear dependency (Figure 4B). We note that the fluorescent assays are measured with a lower LPR and on much longer time-scales (minutes rather than seconds) and hence in these experiments, multiple turnovers of MntH2 give rise to the expected Michaelis–Menten kinetics.

If the magnitudes of the electrophysiology signals are limited by a thermodynamic equilibrium and not by MntH2 kinetics, this equilibrium could either be generated by a equalisation of Mn(II) concentration in the lumen or by the generation of a sufficiently high electrochemical potential (Δψ) to fully oppose further Mn(II) transport [25]. Here, we propose the latter. The electrophysiology experiments cannot be performed in the presence of valinomycin as this not only dissipates Δψ, but also the signal. Hence, it is proposed a Δψ is rapidly established and this opposes further uptake of metal.

Interestingly, Ni(II) uptake was clearly established with fluorescent assay, but uptake signals were absent with the electrophysiology assay. Unexpected is also that no uptake signals are observed with Fe(II) in our electrophysiology assays. Expression of *S. faecalis* MntH2 in *E. coli* has previously been shown to enhance its sensitivity towards Co(II) and Cd(II), but not Fe(II), which might suggest that Fe(II) is not transported by MntH2 [26]. Interestingly, the latter study also expressed *S. faecalis* MntH in *E. coli* and unlike MntH2, expression of MntH did increase sensitivity to Fe(II). Still, it seems unlikely that Fe(II) is not transported by MntH2. The Nramp homologue in *S. capitis* was shown to bind Fe(II) in crystallography [16], while the Nramp homologue from *D. radiodurans* was shown to take up Fe(II) when heterogeneously expressed in *E. coli* [17]. Both these homologues share a high identity with MntH2 (67 and 33% for *S. captitis* and *D. radiodurans*, respectively) and all the amino acids in the metal-binding site are conserved in MntH2. Other members of the Nramp family, including human divalent metal-ion transporter-1 (DMT1) have also been shown to take up Fe(II) [27]. Cu(II), Ni(II) and Fe(II) abolish the electrophysiology signals of Mn(II) uptake, indicating that they compete with Mn(II) and thus that they bind the same metal site, as expected. The electrophysiology data could be explained if the uptake of Fe(II) and Ni(II) is electroneutral, but this would require co-transport of hydroxide ions or counter transport of protons. Although unlikely, proton transport has been known to be decoupled in Nramp transporters under certain conditions (vide infra). Alternatively, the absence of a signal could be explained if the kinetics are slower as the current in electrophysiology is proportional to charge transport kinetics. However, as explained above, the data suggest that the peak currents are not limited by uptake kinetics, at least for Mn(II).

In spite of a strong pH dependence, our electrophysiology experiments could not provide direct evidence that protons are co-transported. This might suggest that MntH2 is a uniporter under the conditions used here (Mn(II) transport and pH > 7) and it is possible that transport by MntH2 at pH > 7.0 is driven by Δψ. Courville et al. studied Cd(II) uptake in right-side out vesicles (RSOV) from *E. coli*, expressing its native MntH. In the presence of nigericin, which abolishes ΔpH but maintains Δψ, Cd(II) transport was increased. In contrast, Cd(II) transport was abrogated by valinomycin, which collapses the Δψ. This suggests that *E. coli* MntH does not rely on ΔpH but is dependent on Δψ [25]. Nonetheless, these results by Courville et al. do not explain why no proton symport is observed in *E. faecalis* MntH2. Even if the Δψ would be sufficient to drive metal uptake by *E. faecalis* MntH2, proton symport could still contribute to the driving force of uptake. Proton co-transport is known to vary between members of the Nramp family and does not always follow a strict stoichiometric ratio, but depends on the condition such as pH and even the metal substrate [28,29,30]. Functional studies on Nramp highlighted several conserved residues in close vicinity of the metal-binding pocket that are important for the proton-coupled aspect of the transport mechanism [15,17,30,31]. Notably, mutating a conserved histidines to an alanine in Nramp from *S. capitis* (His236) or human (His272) retains their ability to transport manganese, but proton transport was abolished [15,29]. This correlates with studies on *E. coli* MntH, in which the LeuT motifs in transmembrane (TM) helices 1 (Asp-Pro-Gly) and 6 (Met-Pro-His) were found to be key to the metal-proton symport activity (the histidine in the Met-Pro-His motif aligns with the *S. capitis* (His236) and human (His272) Nramp) [25]. In stark contrast, however, mutating the homologue histidine in *D. radiodurans* (His237) locks the protein in an outward closed state, rendering the transporter inactive [31]. Furthermore, other *D. radiodurans* MntH mutants, which lock the transporter into either an inside- or outside-open state, show proton transport in the locked outside-open state (without metal transport), but not the inside-open state. This suggests that proton and metal transport have two separate pathways or mechanisms and proton transport does not require large-scale conformational changes. In an Nramp transporter in yeast, Fe(II) uptake becomes less coupled to proton transport when the transmembrane electrochemical potential is increased from −20 to −80 mV [28]. Similarly, in an Nramp from rat (SLC11A2), Fe(II) uptake is coupled to proton transfer at pH_o_ 5.5, but not at pH_o_ 7.4 [29]. Together, all these findings indicate that proton coupling is highly variable between members of the Nramp family and can be dependent on conditions and this could explain why evidence is found for proton co-transport with MntH2 at pH > 7. An interesting observation here is that the current signals diminish when the pH is lowered to pH 6.0. Although this could simply reflect a pH optimum of MntH2, it is possible that at more acidic pH, proton symport is required, but that in the absence of a pH gradient, the electrophysiology signal diminishes. More studies are required to study the pH effect. Whether proton uncoupling, ‘slips’ or leaks are non-ideal properties of membrane proteins or an evolutionary designed feature in biology is under debate [32,33].
